# Evaluating the effectiveness of early urethral catheter removal combined with intermittent catheterization for promoting early recovery of bladder function after laparoscopic radical hysterectomy: a study protocol for a randomized controlled trial

**DOI:** 10.1186/s13063-024-08266-8

**Published:** 2024-06-28

**Authors:** Yanli Chen, Ling Li, Yuanxiang Shi, Xin Rong, Yan Wang, Jiaojiao Wu, Xiaolong Liang, Zhimin Wu

**Affiliations:** Department of Gynecology, the First Affiliated Hospital (Southwest Hospital), Army Medical University of PLA (Third Military Medical University), Chongqing, 400038 China

**Keywords:** Radical hysterectomy, Intermittent catheterization, Indwelling catheters, Bladder function, Randomized controlled trial

## Abstract

**Background:**

Bladder dysfunction, notably urinary retention, emerges as a significant complication for cervical cancer patients following radical hysterectomy, predominantly due to nerve damage, severely impacting their postoperative quality of life. The challenges to recovery include insufficient pelvic floor muscle training and the negative effects of prolonged postoperative indwelling urinary catheters. Intermittent catheterization represents the gold standard for neurogenic bladder management, facilitating bladder training, which is an important behavioral therapy aiming to enhance bladder function through the training of the external urethral sphincter and promoting the recovery of the micturition reflex. Nevertheless, gaps remain in current research regarding optimal timing for intermittent catheterization and the evaluation of subjective symptoms of bladder dysfunction.

**Methods:**

Cervical cancer patients undergoing laparoscopic radical hysterectomy will be recruited to this randomized controlled trial. Participants will be randomly assigned to either early postoperative catheter removal combined with intermittent catheterization group or a control group receiving standard care with indwelling urinary catheters. All these patients will be followed for 3 months after surgery. The study’s primary endpoint is the comparison of bladder function recovery rates (defined as achieving a Bladder Function Recovery Grade of II or higher) 2 weeks post-surgery. Secondary endpoints include the incidence of urinary tract infections, and changes in urodynamic parameters, and Mesure Du Handicap Urinaire scores within 1 month postoperatively. All analysis will adhere to the intention-to-treat principle.

**Discussion:**

The findings from this trial are expected to refine clinical management strategies for enhancing postoperative recovery among cervical cancer patients undergoing radical hysterectomy. By providing robust evidence, this study aims to support patients and their families in informed decision-making regarding postoperative bladder management, potentially reducing the incidence of urinary complications and improving overall quality of life post-surgery.

**Trial registration:**

ChiCTR2200064041, registered on 24th September, 2022.

## Administrative information

Note: the numbers in curly brackets in this protocol refer to SPIRIT checklist item numbers. The order of the items has been modified to group similar items (see http://www.equator-network.org/reporting-guidelines/spirit-2013-statement-defining-standard-protocol-items-for-clinical-trials/).

**Table:** 

Title {1}	Evaluating the effectiveness of early urethral catheter removal combined with intermittent catheterization for promoting early recovery of bladder function after laparoscopic radical hysterectomy: A study protocol for a randomized controlled trial
Trial registration {2a and 2b}.	ChiCTR2200064041,registered on 24th September,2022 https://www.chictr.org.cn/index.html
Protocol version {3}	Version 2.0, 15th October,2021
Funding {4}	N/A
Author details {5a}	Yanli Chen, E-mail: cyl_xnyy@163.com. Department of Gynecology, the First Affiliated Hospital (Southwest Hospital) of Army Medical University of PLA (Third Military Medical University), Chongqing 400,038, China. Ling Li, E-mail: 22,024,896@qq.com. Department of Gynecology, the First Affiliated Hospital (Southwest Hospital) of Army Medical University of PLA (Third Military Medical University), Chongqing 400,038, China. Yuanxiang Shi, E-mail: 990,540,256@qq.com. Department of Gynecology, the First Affiliated Hospital (Southwest Hospital) of Army Medical University of PLA (Third Military Medical University), Chongqing 400,038, China. Xin Rong, E-mail: 401,046,426@qq.com. Department of Gynecology, the First Affiliated Hospital (Southwest Hospital) of Army Medical University of PLA (Third Military Medical University), Chongqing 400,038, China. Yan Wang, E-mail: 401,046,426@qq.com. Department of Gynecology, the First Affiliated Hospital (Southwest Hospital) of Army Medical University of PLA (Third Military Medical University), Chongqing 400,038, China. Jiaojiao Wu, E-mail: 42,397,614@qq.com. Department of Gynecology, the First Affiliated Hospital (Southwest Hospital) of Army Medical University of PLA (Third Military Medical University), Chongqing 400,038, China. ^*^corresponding author: Xiaolong Liang, E-mail: 283,785,027@qq.com. Department of Gynecology, the First Affiliated Hospital (Southwest Hospital) of Army Medical University of PLA (Third Military Medical University), Chongqing 400,038, China. ^*^corresponding author: Zhimin Wu, E-mail: wzm_xnyy@126.com. Department of Gynecology, the First Affiliated Hospital (Southwest Hospital) of Army Medical University of PLA (Third Military Medical University), Chongqing 400,038, China.
Name and contact information for the trial sponsor {5b}	This is an investigator-initiated clinical trial conducted by independent investigators. Yanli Chen (cyl_xnyy@163.com) is the lead investigator.
Role of sponsor {5c}	This is an investigator-initiated study and the sponsor is involved in study design, data collection and analyses as well as manuscript preparation.

## Introduction

### Background and rationale {6a}

Cervical cancer remains a significant challenge to global public health. According to the WHO’s International Agency for Research on Cancer (IARC) 2020 data [1], there were 604,127 new cases of cervical cancer and 341,831 deaths worldwide. Radical hysterectomy (RH), coupled with pelvic lymphadenectomy, is standard treatment for early-stage cervical cancer [2, 3]. However, this surgical intervention often results in bladder dysfunction due to nerve damage, leading to impaired urination control and neurogenic bladder (NB) [4, 5]. The reported incidence of post-surgical bladder dysfunction varies widely, ranging 12 to 85% [6–8]. Symptoms include urinary retention, dysuria, incomplete urination, and urinary incontinence, with urinary retention being the most prevalent [9]. These symptoms elevate the risk of serious complications such as postoperative urinary tract infections, hydronephrosis, bladder rupture, increased rates of outpatient visits, and hospital readmissions [10, 11]. Factors contributing to impaired recovery of bladder function include insufficient pelvic floor muscle training and prolonged postoperative indwelling urinary catheters (IDUCs), which affect the bladder’s natural filling and emptying processes.

Clinical studies have demonstrated that the potential for full restoration of bladder function within 9–12 months post-surgery is through comprehensive lower urinary tract management, focusing particularly on the bladder [6]. Postoperative bladder dysfunction management includes pharmacological treatments such as acetylcholine release stimulants and α-adrenergic receptor blockers, as well as non-pharmacological interventions like intermittent catheterization (IC) and bladder training, which are noted for their simplicity, minimal invasiveness, and low side effects [5]. IC, the periodic emptying of the bladder using a catheter, is supported by class A evidence in NB management and is considered the gold standard [12]. Bladder training, a behavioral therapy aiming to improve bladder function and establish regular urination, has proven effective in preventing post-RH bladder dysfunction and is recommended in nursing practice guidelines for NB management [13]. While there is no consensus on the optimal time for IDUCs removal post-RH, the typical practice varies from 3 to 14 days influenced by hospital policy, surgeon preference, and experience, with potential delays in removal [14, 15]. Some studies advocate for IC to post-catheter removal to manage urinary retention maintaining moderate intravesical pressure to minimize complications, and improve both bladder function rehabilitation and patient quality of life [12, 16].

Despite retrospective studies have compared the application effectiveness of early postoperative catheter removal combined with IC for bladder function recovery [17–20], these studies lack the assessment of subjective symptoms of bladder dysfunction in cervical cancer patients post-RH. This highlights the need for further high-quality randomized controlled trials (RCT) to reassess these strategies. Therefore, this study intends to compare early urinary catheter removal (3 days post-surgery) followed by IC in patients with grade II or lower bladder function recovery against conventional IDUCs approaches in a randomized parallel-controlled trial. By establishing a reasonable hydration plan to stabilize urine production, this trial aims to facilitate bladder training and promote urination reflex recovery. The primary objective of this study is to evaluate the effectiveness and safety of this intervention, offering a new active bladder function management post-RH.

### Objectives {7}

The primary objective of this study is to evaluate the effectiveness and safety of early urinary catheter removal combined with IC in restoring bladder function among patients with cervical cancer post-RH, as compared to standard care with IDUCs.

### Trial design {8}

**Fig. 1:**
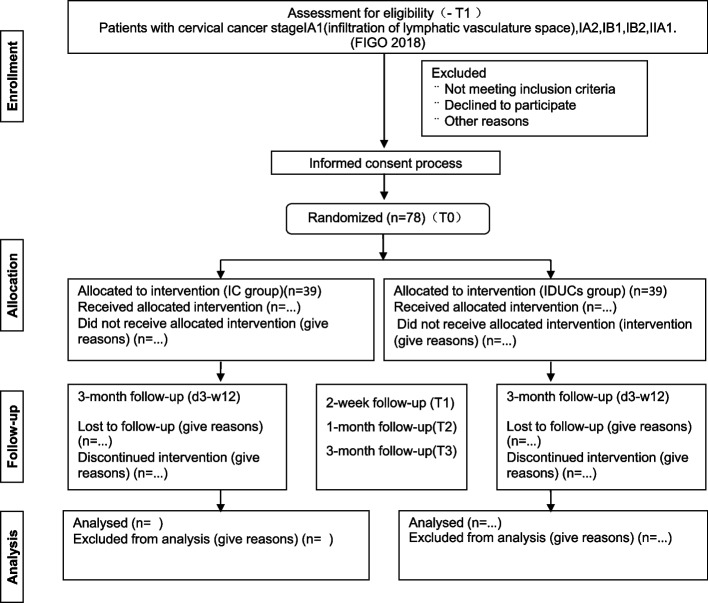
The Flowchart of the study. IDUCs: indwelling urinary catheters, IC: intermittent catheterization

This study is a parallel-group superiority randomized controlled trail conducted at a single center, with an allocation ratio of 1:1. We hypothesize that early urinary catheter removal combined with IC is superior in terms of efficiency and effectiveness compared to IDUCs. All participants in the study will be assessed weekly and followed for 3 months. The flow chart is shown detailing the study design, and all interventions and outcome measures are presented in Fig. 1 and Table 1 respectively. This trial follows the report standard of SPIRIT 2013.

**Table 1.**
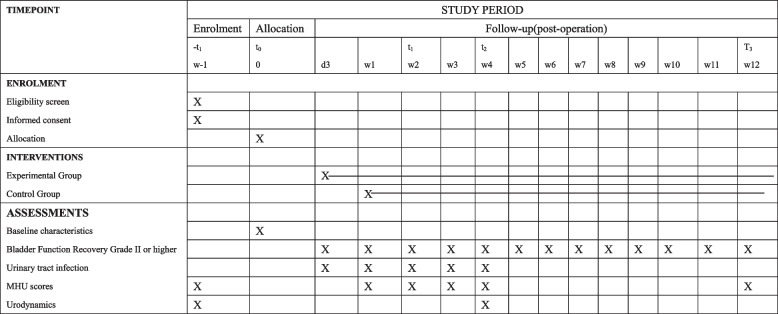
Enrolment and outcome measurements

## Methods: participants, interventions, and outcomes

### Study setting {9}

All participants are recruited from the First Affiliated Hospital of Army Medical University of PLA in Chongqing, China. The Ethics Committee of this hospital has approved this study protocol, (Approval No.(A)KY2021113)). All potential participants will sign a written informed consent form before allocation.

### Eligibility criteria {10}

#### Inclusion criteria

- →Prospective enrolment of patients diagnosed with cervical cancer stages IA1 (infiltration of lymphatic vasculature space), IA2, IB1, IB2, and IIA1, according to the FIGO 2018 classification [21].
- →Patients underwent Laparoscopic RH and pelvic lymphadenectomy.
- →Aged 20 to 65 years, and BMI < 30 kg/m^2^.
- →Classified as I or II under the American Society of Anesthesiologists (ASA) classification.
- →A score of more than 75 points on the preoperative Barthel self-care ability assessment.

#### Exclusion criteria

- →Patients with pre-existing urinary dysfunction diseases, kidney diseases, or serious underlying conditions, such as uncontrolled severe anemia, hypertension, diabetes, and cardiovascular or cerebrovascular complications.
- →Surgical duration exceeding 4 h, or the blood loss surpassing 1000 mL.
- →Intraoperative injuries to the ureter, bladder, or bowel; Postoperative complications such as vesicovaginal or urethrovaginal fistulas
- →Conditions requiring emergency intervention or reoperation within 6 h post-operation
- →Patients with severe mental illness, or cognitive dysfunction.→

#### Exit criteria

- →Participants are reluctant to continue the study.
- →If researchers believe that continued participation would be detrimental to patient treatment.

### Who will take informed consent? {26a}

Patients meeting the above inclusion criteria will be informed about this study after admission. Interested patients will attend an orientation session and be allocated a handbook regarding postoperative bladder management before surgery. Participants, who can review and complete consent independently will then be provided with the informed consent form. Those who agree to participate will sign a written informed consent form prior to enrollment.

### Additional consent provisions for collection and use of participant data and biological specimens {26b}

No biological specimens will be collected. No additional participant data beyond what is outlined will be utilized.

## Interventions

### Explanation for the choice of comparators {6b}

In the control group, catheters will be removed seven days post-operation as determined by the attending physician. If residual urine volume (RUV) exceeds 100 mL upon catheter removal, this will indicate urinary retention, promoting reinsertion of the catheter. The catheter will be removed on a weekly basis thereafter, until RUV is less than 100 mL [22–24].

### Intervention description {11a}

1. Health education (preoperative): Prior to the surgery, patients and their family members will be provided with education on IC. This will include an overview of the principles, methodology, and procedural steps of IC, complemented by instructional videos.
2. Supervision and guidance (postoperative days 1–2): Study team members will provide supervision and guidance to patients regarding pelvic floor muscle training (PFMT). Continued education on IC will be delivered through instructional videos to patients and their families.
3. Catheter removal process (postoperative day 3): Following the prescribed fluid intake plan, IDUCs will be removed according to the physician’s instructions. Patients will be encouraged to urinate spontaneously 2–3 times within 2 h of catheter removal. After the last voiding, RUV will be immediately assessed via ultrasound. If RUV exceeds 100 mL, IC will be initiated. The frequency of IC will be adjusted based on RUV. When the frequency of catheterization decreases to once per day and RUV is less than 100 mL after spontaneous urination, bladder voiding function will be considered to be recovered to level II or higher.
4. Patient ability to perform IC will be evaluated by the pencil and paper test [25]. The total score is 15 points, with a score ≥ 9 points indicating patient can perform IC with assistance from medical staff or family. Those scoring < 9 points will require direct assistance from medical staff.
5. Scheduling of IC: The frequency of IC will be tailored according to RUV measurements: 5–6 times daily for RUVs of 350–500 mL (5 times per day: 7:00, 11:00, 15:00, 19:00, 22:00; 6 times per day: 7:00, 11:00, 15:00, 19:00, 22:00, 1:00); 3–4 times daily for RUVs of 200–350 mL (3 times per day: 7:00, 13:00, 19:00; 4 times per day: 7:00, 13:00, 19:00, 22:00); 1–2 times daily for RUVs of 100–200 mL (once per day at 19:00; twice per day at 7:00 and 19:00). Specific timing for catheterization is outlined.
6. Drinking plan: Total daily fluid intake is set 1500–2000 mL, and not exceed 2500 mL [12, 13]. Drink 400–500 mL of each meal (including intravenous infusion, liquid beverages, milk, soup). Between meals, patients may drink 200–300 mL of fluids every time. Fluids should be avoided from 20:00 to 6:00.
7. Voiding diary for IC: Participants will record intake amounts (including intravenous infusion volume during hospitalization), spontaneous voided volume, urination times, IC interval, catheterized volume, uresiesthesia and symptoms over a continuous 24-h period. Milliliter was used for fluid unit. And fluid volume was determined by professional tools.
8. Bladder training: Exception pelvic floor rehabilitation training, patients undergoing IC will participate in bladder training, which includes scheduled urination, urge suppression, and compensatory voiding maneuvers to enhance urinary behavior.

### Criteria for discontinuing or modifying allocated interventions {11b}

Patients may switch intervention options or withdraw from this study at their discretion. If a patient decides to withdraw, efforts will be made to finalize and document their data beforehand. The reasons and timing of patient withdrawal will be detailed in the case report. If patients opt out of the trial, their study participation may be terminated.

### Strategies to improve adherence to interventions {11c}

A team member will be assigned to manage each participant’s follow-up through WeChat APP or telephone post-discharge. And participants will submit their “IC Voiding Diary (intervention group)” and “Indwelling Catheter Care Record (control group)” to research team, enhancing adherence significantly.

### Relevant concomitant care permitted or prohibited during the trial {11d}

All participants enrolled in the study will receive essential baseline care throughout the study, including pelvic floor rehabilitation training starting 6 h post-operation and continue for up to 3 months [26].

### Provisions for post-trial care {30}

The trial does not increase risks beyond what may result from routine management. All patients will receive routine postoperative care independent their allocation.

### Outcomes {12}

#### Primary outcomes

The primary outcome measure for this study is the rate of bladder function recovery (Bladder Function Recovery Grade II or higher) 2 weeks post-surgery. Bladder function recovery is quantified by a RUV less than 100 mL following spontaneous urination after catheter removal. The grading system for bladder function recovery is as follows [27]: Grade I: Patient has a bladder fullness sensation and urination desire, can urinate spontaneously upon catheter removal, and has a RUV of less than 50 mL, indicating effective bladder function recovery; Grade II: Patient has a bladder fullness sensation and the desire to urination, can urinate spontaneously upon catheter removal, but the RUV is between 50 and 100 mL, suggesting slightly diminished bladder function recovery. Grade III: Patient lacks bladder fullness sensation or urination desire, can urinate spontaneously upon catheter removal, but RUV exceeds 100 mL, reflecting poor bladder function recovery; Grade IV: Patient has no sensation of spontaneous urination and cannot urinate independently with various interventions after catheter removal, indicating lack of bladder function recovery.

#### Secondary outcomes

1. Urinary tract infection within the first month post-surgery [28]. Patients have symptoms, including urinary urgency, frequency, pain, or other urinary irritation symptoms, as well as lower abdominal tenderness, flank tenderness, and potentially with or without fever. Diagnosis is confirmed by finding white blood cells ≥ 10/ high power field in urine examinations, coupled with urine cultures in catheterized patients.
2. Bladder dysfunction within three months post-surgery: Assessed using the Mesure Du Handicap Urinaire (MHU) bladder function scale for self-assessment.
3. Changes in urodynamic parameters within 1 month post-surgery: This involves comparing urodynamic measurements, including maximum flow rate, average flow rate, RUV, bladder safe capacity, maximum detrusor pressure, and bladder compliance.

### Participant timeline {13}

The timeline for participant was detailed in Table 1.

### Sample size {14}

Based on the bladder function recovery rate at 2 weeks post-surgery (Bladder Function Recovery Grade II or higher) observed in pretest, the sample size was determined. The recovery rate for the IC group was 78.95%, compared to 38.89% IDUCs group. Using a unilateral *α* of 0.025, a desired power of 0.9 (i.e., 1 − *β* = 0.9) and the margin of 5%, the sample size was calculated using the following formula.

$$n=\frac{{2\overline{p }\overline{q }\left({z}_{\alpha }+{z}_{\beta }\right)}^{2}}{{\left({p}_{1}-{p}_{2}\right)}^{2}}$$

Based on these parameters, the calculated sample size for each group was determined to be 35 cases. To facilitate 1:1 randomization, a total of 70 cases would be required. However, anticipating potential loss due to follow-up dropouts and refusal of participation (estimated at 10–15%), the final minimum number of research subjects for each group was determined to be 39 cases. Therefore, at least 78 participants are deemed necessary to ensure sufficient statistical power for this study.

### Recruitment {15}

Patient recruitment for the study will follow a consecutive enrollment strategy at the First Affiliated Hospital of Army Medical University. Recruitment was initiated in September 26th 2022. Due to the COVID-19, it is expected to be finished by the end of 2024. Participants were recruited by two strategies. Patients from outpatient and inpatient, meeting inclusion criteria, were referred to project researchers for recruitment. Eligible hospitalized patients were recruited. The estimated recruitment fraction is 40%. Seven patients will be recruited per month, and 2–3 of them will be enrolled in this trial.

## Assignment of interventions: allocation

### Sequence generation {16a}

As a prospective randomized controlled design, the intervention group and the control group were designed in a 1:1 ratio. With the rank function of the computer EXCEL table to generate 78 random real numbers, sort and group the random data, and participants are grouped accordingly: individuals assigned odd numbers join the intervention group, while even numbers denote control group membership.

### Concealment mechanism {16b}

The random assignment sequences are generated and managed by an individual not involved in clinical duties ensuring that the allocation remains concealed. This is achieved through the use of sequentially numbered and sealed opaque envelopes, which are then distributed randomly to participants.

### Implementation {16c}

Upon signing the consent form, participants are allocated to either the intervention or control group according to numbers in the envelope.

## Assignment of interventions: Blinding

### Who will be blinded {17a}

Given the nature of intervention, it is challenging to blind participants and staff to group allocation. However, allocation status of participants is kept hidden from those evaluating the outcomes. To minimize potential bias, all outcomes not based on self-reporting will be assessed by an evaluator who is unaware of the participants’ treatment allocation.

### Procedure for unblinding if needed {17b}

N/A: Only outcome evaluators are blinded in this study. So unblinding does not apply to this study’s design.

## Data collection and management

### Plans for assessment and collection of outcomes {18a}

Outcome assessments will be meticulously documented by the research evaluator on the case report form (CRF), identifying participants by their study ID numbers and initials, thus ensuring confidentiality. Baseline assessments will be conducted prior to surgery. Evaluations for postoperative urinary tract infection are scheduled at 3 days, 1 week, 2 weeks, 3 weeks, and 4 weeks post-surgery. Assessments for bladder dysfunction post-operation will occur at 1 week, 2 weeks, 3 weeks, 1 month, and 3 months post-surgery. Urodynamic results will be collected 1 month post-surgery. Follow-up will be conducted through WeChat APP or telephone.

### Plans to promote participant retention and complete follow-up {18b}

To enhance participant retention and ensure comprehensive follow-up, individuals will be informed about the study’s design, its significance, and the potential benefits of participation during the recruitment phase. Each participant has an assigned a research member responsible for data collection and management. Participants will be informed of their right to alter intervention options or withdraw from the study. And all the information and results of these participants will be collected with permission.

### Data management {19}

All collected data will be securely stored with backups created, and access to the data will be restricted. Once all participants have completed their follow-up and the data is verified, the primary investigator will lock the raw data to prevent further changes. All study-related materials will be retained by the Research Office of the First Affiliated Hospital of Army Medical University. Data will be available to professional statisticians for statistical analysis while maintaining the integrity of the raw data files for future reference.

### Confidentiality {27}

Participants’ privacy and confidentiality will be rigorously protected. All personal data will be stored securely with limited access. The identification details of participants will not be utilized in any aspect of the research dissemination.

### Plans for collection, laboratory evaluation, and storage of biological specimens for genetic or molecular analysis in this trial/future use {33}

N/A: This study does not involve the collection, evaluation, or storage of biological specimens for genetic or molecular analysis.

## Statistical methods

### Statistical methods for primary and secondary outcomes {20a}

An intention-to-treat analysis will be performed, utilizing the last available measurement for instance of data lost. The collected data was analyzed using SPSS 20.0 software. Continuity data will undergo normality testing; those with a normal distribution will be analyzed using an independent sample *t* test, while those with a skewed distribution will be assessed using non-parametric test. Chi-square test was used for enumeration data, mean value ± standard deviation ( $\overline{x }$±S) for measurement data. When *p* < 0.05, the difference can be considered statistically significant.

### Interim analyses {21b}

N/A: No detrimental problems to patients enrolled in this study are anticipated. Official interim analysis of the primary and secondary outcomes is not applicable in this study.

### Methods for additional analyses (e.g., subgroup analyses) {20b}

N/A: The primary analysis is sufficient to address the research question. No additional analyses were required.

### Methods in analysis to handle protocol non-adherence and any statistical methods to handle missing data {20c}

The intention-to-treat analysis will be used in this study. Missing objective data will be interpolated with mean values, and the missing subjective data will not be interpolated. The effectiveness of these methods for handling missing data will be assessed through sensitivity analysis. Further, to evaluate the impact of non-adherence on study outcomes, the per-protocol analysis will also be adopted.

### Plans to give access to the full protocol, participant-level data, and statistical code {31c}

The full protocol, participant-level data, and statistical code are available from the corresponding author upon reasonable request.

## Oversight and monitoring

### Composition of the coordinating center and trial steering committee {5d}

This is a single-center study. The independent investigator will be in charge for the management, and correspondence authors will meet and discuss with research team members weekly. And there is no steering committee.

### Composition of the data monitoring committee, its role and reporting structure {21a}

The data monitoring committee (DMC) includes clinicians and statisticians, independent of the study team. It monitors the safety and ensures the data quality of this study.

### Adverse event reporting and harms {22}

While specific risks to participants are not expected to increase, adverse reactions may occur. Possible risks of the study include complications such as severe urinary retention, urinary tract infections, and hydronephrosis. All adverse events will be recorded. In case of serious adverse events with risk to the patient’s life, it must be reported to the ethics committee within 24 h. Their relevance of these events to this study will be determined, and participants will be compensated for any damage caused by the study participation, in accordance with relevant laws and regulations.

### Frequency and plans for auditing trial conduct {23}

The management team will meet twice a month to assess study progress and address any arising issues. Due to the low risk of intervention, no audit for trail conduct is anticipated. Ethics Committee meet to review this trial once a year.

### Plans for communicating important protocol amendments to relevant parties (e.g., trial participants, ethical committees) {25}

No changes will be made to informed consent, recruitment materials, and other related documents without prior review and approval by the ethics committee. If necessary, the revised protocol must be updated in the Chinese Clinical Trial Registry center. And all interested parties will be informed.

### Dissemination plans {31a}

Study results will be disseminated through public presentations and publication in medical peer-reviewed journals. Report of this study will be submitted to Chinese Clinical Trial Registry center.

## Discussion

Bladder dysfunction (urinary retention) after RH is generally defined as the inability to urinate spontaneously for more than 14 days after surgery, or the RUV > 100 mL although it is able to urinate spontaneously [24]. Therefore, the primary outcome of this study was to determine the rate of recovery of bladder function at 2 weeks postoperatively. This trial aims to assess whether patients will benefit from this new intervention. Firstly, this study avoids the prolongation of the catheterization for patients who have recovered bladder function early. Secondly, for patients with insufficient recovery of bladder function (below grade II) after catheter removal, IC can be initiated, providing an opportunity for bladder training, including regular spontaneous and conscious urination. Additionally, it is convenient to preliminarily evaluate the recovery of bladder function according to the self-urination volume. Thirdly, the early removal of urethral catheter post-surgery reduces the indwelling time of the catheter, lowers the incidence of urinary tract infection, and improves the comfort of patients. This combination of IC and bladder training to promote early bladder function rehabilitation in patients with cervical cancer surgery has been rarely reported [17, 18, 20]. Therefore, we intend to evaluate the feasibility and effectiveness of this intervention in the management of the bladder after cervical cancer post-RH through RCT. The results will provide reliable evidence and support for the early recovery of bladder function in patients after radical cervical cancer surgery.

The clinical applicability of this study is reflected in several aspects. The inclusion criteria essentially encompass all early-stage cervical cancer patients to promote the generalizability of evidence to a broad population. Meanwhile, the narrow exclusion criteria merely ensure safety for subjects undergoing intervention. Another practical aspect of this study is the execution of IC, which can be implemented by healthcare professionals, the patients themselves, or their family members. Additionally, regardless of subject compliance, primary outcome includes the experimental results of all subjects, aiming to determine whether intervention is effective under normal conditions.

The intervention, standard procedure for IC, is relatively easy to perform, but adjusting the frequency of IC is more complex. Typically, the frequency of catheterization is based on the RUV. Regular bladder filling and emptying contribute to bladder function recovery. In this study, personalized drinking plans are designed as intervention. By guiding patients to record urination diaries and identifying the pattern of urine production, the principle of input equals output is applied, with the bladder’s safe capacity as a standard. Personalized drinking plans are developed based on the patient’s dietary habits and daily routines to affect urine production and guide urination frequency. The drinking plans should fully consider the patient’s lifestyle habits and make corresponding adjustments based on factors such as sweating and weather changes to avoid urinary tract damage caused by excessive hydration exceeding the bladder’s safe capacity. Furthermore, patient compliance is also a challenge for quality control. To ensure that participants fully understand the benefits of participating in this study, we have developed relevant videos and educational manuals that can be accessed through mobile phones, and computers. This greatly facilitates patients in addressing issues encountered during the bladder function recovery process. We provide one-on-one services to each patient who has enrolled in this clinical trial via WeChat apps and phone calls to improve patient compliance and reduce data loss.

The strength of this trail lies in its capacity to provide high-level evidence for informed healthcare decisions by patients and their families with significant clinical and practical implications for understanding bladder function recovery process and strategies. This, in turn, promotes patient’s rehabilitation and quality of life.

However, this study has certain limitations. Being conducted at a single center, the findings’ generalizability may be constrained by the specific expertise and experience of medical staff at this center. Additionally, due to the nature of the interventions, which necessitate patient participation, it is not feasible to blind researchers and subjects to group allocations, potentially introducing bias.

## Trial status

The protocol version is Version 2.0 of Oct. 15th, 2021. The trial protocol is registered as ChiCTR2200064041 in the Chinese Clinical Trial Registry on Sept 24th, 2022.The first patient was recruited on Sept 26th, 2022, and the study is supposed to be completed by Dec 31st, 2024.

## Data Availability

De-identified data in the current study will be available upon reasonable request from the corresponding author.

## Abbreviations

DMC: Data monitoring committee
IARC: International Agency for Research on Cancer
IC: Intermittent catheterization
IDUCs: Indwelling urinary catheters
RH: Radical hysterectomy
MHU: Mesure Du Handicap Urinaire
NB: Neurogenic bladder
PFMT: Pelvic floor muscle training
RUV: Residual urine volume
